# Induced pluripotent stem cells reprogrammed from primary dendritic cells provide an abundant source of immunostimulatory dendritic cells for use in immunotherapy

**DOI:** 10.1002/stem.3095

**Published:** 2019-10-31

**Authors:** Christopher Horton, Timothy J. Davies, Priyoshi Lahiri, Patty Sachamitr, Paul J. Fairchild

**Affiliations:** ^1^ Sir William Dunn School of Pathology University of Oxford Oxford United Kingdom

**Keywords:** dendritic cell, epigenetic memory, immunostimulation, immunotherapy, induced pluripotent stem cell, maturation

## Abstract

Cell types differentiated from induced pluripotent stem cells (iPSCs) are frequently arrested in their development program, more closely resembling a fetal rather than an adult phenotype, potentially limiting their utility for downstream clinical applications. The fetal phenotype of iPSC‐derived dendritic cells (ipDCs) is evidenced by their low expression of MHC class II and costimulatory molecules, impaired secretion of IL‐12, and poor responsiveness to conventional maturation stimuli, undermining their use for applications such as immune‐oncology. Given that iPSCs display an epigenetic memory of the cell type from which they were originally derived, we investigated the feasibility of reprogramming adult DCs to pluripotency to determine the impact on the phenotype and function of ipDCs differentiated from them. Using murine bone marrow‐derived DCs (bmDCs) as proof of principle, we show here that immature DCs are tractable candidates for reprogramming using non‐integrating Sendai virus for the delivery of Oct4, Sox2, Klf4, and c‐Myc transcription factors. Reprogramming efficiency of DCs was lower than mouse embryonic fibroblasts (MEFs) and highly dependent on their maturation status. Although control iPSCs derived from conventional MEFs yielded DCs that displayed a predictable fetal phenotype and impaired immunostimulatory capacity in vitro and in vivo, DCs differentiated from DC‐derived iPSCs exhibited a surface phenotype, immunostimulatory capacity, and responsiveness to maturation stimuli indistinguishable from the source DCs, a phenotype that was retained for 15 passages of the parent iPSCs. Our results suggest that the epigenetic memory of iPSCs may be productively exploited for the generation of potently immunogenic DCs for immunotherapeutic applications.

## Graphical abstract

The phenotype and function of dendritic cells (DCs) differentiated from induced pluripotent stem cells (iPSCs) is profoundly influenced by the cell type of origin. iPSCs reprogrammed from mouse embryonic fibroblasts yield poorly immunogenic DCs, consistent with a “fetal” phenotype. In contrast, reprogramming of immature mouse DCs to pluripotency captures an epigenetic memory that supports the differentiation of highly stimulatory DCs, suitable for downstream immunotherapy.

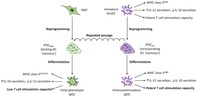

**Significance statement**

Induced pluripotent stem cells (iPSCs) offer a scalable source of rare dendritic cell (DC) subsets, not otherwise accessible from patients, that are amenable to manufacture and quality control. However, in common with other cell types differentiated from a pluripotent source, DCs become arrested in their developmental pathway, significantly limiting their immunogenicity. This study showed that a simple modification to protocols for the derivation of iPSC lines fully resolves this issue and demonstrated the feasibility of reprogramming existing DCs to pluripotency, thereby exploiting the epigenetic memory of iPSCs for the cell type of origin. By capturing the epigenetic profile of existing DCs, the resulting iPSCs spawn populations of DCs that are highly immunostimulatory and suitable for use in immunotherapy.

## 1 INTRODUCTION

As professional antigen‐presenting cells, dendritic cells (DCs) are uniquely responsible for initiating all immune responses and are, therefore, attractive candidates for use in immunotherapy.^1^ Accordingly, DCs differentiated from peripheral blood monocytes (moDCs) have been used in more than 200 clinical trials in an attempt to vaccinate cancer patients to defined tumor associated antigens (TAAs).^2^ Although such trials have demonstrated only limited efficacy, induced pluripotent stem cells (iPSCs) may provide an unlimited source of previously inaccessible subsets of DCs, such as plasmacytoid DCs^3^ and CD141^+^ DCs displaying superior capacity for the cross‐presentation of TAAs.^4^ Unlike moDCs, such a novel source is amenable to scale‐up, quality control and genome editing,^3^, ^5^ making it attractive for downstream therapeutic applications.^6^ Nevertheless, iPSC‐derived DCs (ipDCs) appear arrested at a stage of development more reminiscent of a fetal rather than an adult phenotype, a finding common to many cell types differentiated from a pluripotent source, including hepatocytes,^7^ cardiomyocytes,^8^ and forebrain neurons.^9^ Human iPSC‐derived cardiomyocytes, for example, typically display fetal‐type ion channel expression, spontaneous contraction, and fetal‐type electrophysiology,^8^, ^10^ limiting their capacity to integrate into cardiac tissue and electrically couple with adult cardiomyocytes. In the case of DCs, the fetal phenotype is characterized by low MHC class II expression,^5^, ^11^, ^12^ excessive anti‐inflammatory IL‐10 secretion but barely detectable levels of IL‐12 upon ligation of pattern recognition receptors (PRRs), a similar inability to secret IL‐12 having been described previously among human neonatal DCs.^13^, ^14^

Since their advent more than a decade ago,^15^ iPSCs have been demonstrated by numerous laboratories to display “epigenetic memory” of the cell type from which they were originally derived,^16^, ^17^ attributed to residual DNA methylation patterns and histone modifications within the genome of the source cells.^18^, ^19^, ^20^ Indeed, multiple studies have demonstrated retained memories of cell types of origin within iPSCs, including retinal pigmented epithelial cells,^21^ pancreatic β islet cells,^22^ hepatocytes,^23^ blood progenitors,^16^ granulocytes,^17^ fibroblasts,^16^, ^23^, ^24^ skeletal muscle precursors,^17^ hepatoblasts,^25^ osteoblasts,^26^ melanocytes,^23^ adipocytes,^24^ germline stem cells,^27^ keratinocytes,^24^ bone marrow mesenchymal stem cells,^28^ and cardiac myocytes.^29^ The subtle epigenetic signature of the cell type of origin may contribute to an increased tendency for spontaneous redifferentiation back into the donor cell type^30^: indeed, differentiation of cardiac progenitor cell‐derived iPSCs into cardiomyocytes was shown to be more efficient than fibroblast‐derived iPSCs,^31^ whereas iPSCs derived from mesoangioblasts displayed stronger myogenic commitment than conventional, fibroblast‐derived iPSCs.^32^ This epigenetic memory is, however, eventually lost upon repeated passage of iPSCs,^17^ suggesting that continual cell division progressively removes any epigenetic relicts of past incarnations and promotes resolution of epigenetic differences among iPSC lines.^33^

In order to address the limitations of the fetal phenotype of ipDCs, we investigated the feasibility of reprogramming existing DCs to pluripotency in order to exploit the epigenetic memory of iPSCs for the cell type of origin to facilitate the downstream differentiation of DCs with a definitive, adult phenotype. Given the sentinel function of DCs which render them highly sensitive to viral pathogens and PRR ligands, we anticipated that reprogramming of existing DCs with viral vectors may prove challenging. However, contrary to expectation, we show here that mouse bone marrow‐derived DCs (bmDCs) are fully amenable to reprogramming unless provoked to mature in response to PRR agonists. Furthermore, iPSC lines derived in this way (so‐called iPSC_DC_) show significant capacity for redifferentiation along the DC lineage pathway, the resulting cells displaying superior immunogenicity in vitro and in vivo compared to ipDCs differentiated from conventional iPSC lines derived from MEFs (iPSC_MEF_). Although ultimately lost after passage 15, we conclude that the epigenetic memory retained by the iPSC lines provides an adequate window of opportunity for the production of immunostimulatory DCs for vaccination purposes.

## 2 MATERIALS AND METHODS

### 2.1 Experimental mice

CBA/Ca (H‐2^k^), C57BL/6 (H‐2^b^), and A1.RAG‐1^−/−^ mice (H‐2^k^) were bred and maintained under specific pathogen‐free conditions at the Sir William Dunn School of Pathology (University of Oxford). A1.RAG‐1^−/−^ mice express a transgenic T cell receptor (TCR) (Vα10, Vβ8.1) specific for the male antigen Dby_479‐493_ epitope in the context of H‐2E^k^.^34^ All experimental procedures were conducted in accordance with the Home Office Animals (Scientific Procedures) Act of 1986 and received local ethical committee approval.

### 2.2 Preparation of source DCs

Bone marrow from the dissected femurs of adult male CBA/Ca mice was depleted of erythrocytes and cultured at 7.5 × 10^5^ cells/mL in 90 mm diameter tissue culture plates in R10 medium (RPMI 1640 supplemented with 10% fetal calf serum [FCS], 2 mM L‐glutamine, 1% sodium pyruvate and 50 μM 2‐mercaptoethanol) further supplemented with ~5 ng/mL murine GM‐CSF harvested from the supernatant of an X6310 cell line stably transfected with the murine *Gmcsf* gene. Nonadherent cells were removed on days 3 and 6 of culture when the medium was replaced and cells were harvested on day 7. DCs were purified using anti‐CD11c‐APC monoclonal antibodies (mAb) followed by anti‐APC magnetic beads, according to the manufacturer's instructions (Miltenyi Biotec, Bisley, Surrey, UK).

### 2.3 Derivation of iPSC lines

CD11c^+^ DCs were plated at 1.25 × 10^5^ cells per well of a 96 well plate and reprogrammed using CytoTune‐iPS 2.0 (ThermoFisher Scientific, Loughborough, UK) composed of Sendai virus (SeV) containing the combination of Klf4, Oct4, and Sox2 (KOS) transcription factors or either c‐Myc or Klf4 alone. Multiple conditions were used to identify the optimum ratios of transcription factors for reprogramming including KOS:c‐Myc:Klf ratios of 5:5:3, 20:5:3, 5:5:6, and 5:5:5. Preliminary experiments using mouse embryonic fibroblasts (MEFs) in a side‐by‐side comparison of the ratios 5:5:3 and 5:5:6 resulted in a 10‐fold increase in numbers of iPSC colonies from 29 to 291, respectively, suggesting that increasing the availability of the *Klf4* transgene has a significantly beneficial effect on reprogramming efficiency. These findings were subsequently found to be applicable to the use of bmDCs for reprogramming purposes, a ratio of 5:5:5 yielding substantially more colonies than either 5:5:3 or 20:5:3. The control MEF‐derived iPSC line established previously (iPSC_MEF_SV_2_) was generated using SeV containing Oct4, Klf4, Sox2, and c‐Myc factors. Cell suspensions were incubated with virus overnight after which supernatants were removed daily and replaced with fresh medium. Cells were transferred to six well plates on day 7 containing mitotically inactivated MEF feeder cells. Feeder cells were prepared by incubating MEFs with 10 μg/mL mitomycin C (MMC) in complete medium consisting of Dulbecco's Modified Eagle's Medium (DMEM) supplemented with 10% FCS, 2 mM L‐glutamax, 1.0 mM sodium pyruvate, 100 U/mL penicillin, 100 μg/mL streptomycin (P/S), 0.1 mM nonessential amino acids (NEAAs), and 50 μM 2‐ME for 2‐3 hours. Individual monoclonal iPSC colonies were incubated for 5 days in complete medium further supplemented with 15% FCS and 1000 U/mL recombinant murine Leukemia Inhibitory Factor (rmLIF). Clone iPSC_DC_SV_C_, generated using a ratio of KOS:c‐Myc:Klf4 of 5:5:5 was selected for further use, along with iPSC_MEF_SV_2_. iPSC lines were routinely passaged every 3 days.

### 2.4 Clearance of Sendai viral vectors

In order to assess the clearance of SeV vectors, distinct passages of iPSC_DC_SV_C_ were thawed, established on mitotically inactivated MEFs and passaged on gelatin to remove contaminating fibroblasts. RNA was purified using the PureLink RNA Mini kit (Invitrogen, Loughborough, UK; Cat# 12183018A) according to the manufacturer's instructions, the concentration and purity of the resulting RNA being determined by Nanodrop. Genomic DNA was removed from samples of RNA by mixing with gDNA WipeOut prior to cDNA synthesis using the QuantiTect Reverse Transcription kit (Invitrogen, Cat# 205310). The resulting cDNA (2 μL) was used in a real‐time PCR reaction of 20 μL total volume using the TaqMan Gene Expression assay for SeV, KOS, Klf4, and cMyc (Gene expression assays Mr04269880_mr, Mr04421257_mr, Mr04421256_mr, and Mr04269876_mr, respectively; Life Technologies, Paisley, Scotland, UK, Cat# 4448892) and TaqMan Fast Advanced Master Mix (Life Technologies, Cat# 4444556) according to the manufacturer's instructions. Forty cycles of denaturation at 95°C for 30 seconds, annealing at 55°C for 30 seconds and elongation at 72°C for 30 seconds were performed on an AB Systems 7500 FAST Real‐Time PCR system. Results were analyzed in Excel.

### 2.5 Differentiation of iPSCs

To form embryoid bodies (EBs), iPSC were cultured for two passages on gelatin‐coated flasks to remove feeder cells before being dissociated and plated into bacteriological dishes containing complete medium. To confirm pluripotency, day 14 EBs were grafted under the kidney capsule of CBA/Ca mice. Animals were sacrificed 21 days later; the teratomas were excised and embedded in OCT compound, cryosectioned and stained with hematoxylin (0.5% Harris') and eosin (1%). Sections were viewed using an Olympus CKX41 light microscope and photomicrographs taken using an Olympus SP‐500UZ digital camera. For directed differentiation of DCs, 25‐30 EBs were plated onto 90 mm tissue culture dishes containing differentiation medium (complete medium further supplemented with 5 ng/mL GM‐CSF and 200 U/mL rmIL‐3) as previously described.^35^

### 2.6 Flow cytometry

Cells (2 × 10^5^ per well) were blocked on ice using 1 μL/mL Serobloc in blocking buffer (phosphate‐buffered saline [PBS], 5% heat inactivated normal rabbit serum (HI‐NRS), 1% bovine serum albumin (BSA), 2 mM sodium azide), and stained with anti‐CD11c‐APC (BD Pharmingen, Wokingham, Berks, UK; Cat# 550261), anti‐CD40‐FITC (BD Pharmingen, Cat# 553790), anti‐CD54‐PE (BD Pharmingen, Cat# 553253), anti‐CD80‐PE (BD Pharmingen, Cat# 553769), anti‐CD86‐PE (eBioscience, San Diego, California, Cat# 12‐0861‐81), anti‐H‐2K^k^‐PE (BD Pharmingen, Cat# 553593), anti‐MHC class II‐PE (eBioscience, Cat# 12‐5321‐81) or anti‐SSEA‐1‐PE (eBioscience, Cat# 12‐8813‐71). For analysis of transcription factor expression, cells were permeabilized with 0.1% saponin for 5 minutes and stained using anti‐Nanog‐Alexa Fluor 488 (BD Pharmingen, Cat# 560278) or anti‐Oct4‐Alexa Fluor 647 (BD Pharmingen, Cat# 560329). Samples were washed three times in washing buffer (PBS, 1% FCS, 2 mM NaN_3_), fixed in 2% formaldehyde/PBS, and analyzed using a FACSCalibur cytometer.

### 2.7 Cytokine secretion

DCs differentiated from bone marrow (bmDC) or iPSCs (ipDC) were cultured at 2 × 10^5^ cells per well of 96 well tissue culture plates and stimulated with a serial dilution of lipopolysaccharide (LPS) from *Escherichia coli* Serotype 0127:B8 (Sigma‐Aldrich, Dorset, UK). After 22 hours, culture supernatants were harvested and concentrations of IL‐10 and IL‐12 determined by sandwich ELISA, according to the manufacturer's instructions (eBioscience Mouse IL‐10 [Cat# 88‐7104‐22], IL‐12 p70 [Cat# 88‐7121‐88] ELISA Ready‐SET‐Go! kits). Optical density (OD)_450nm_ and OD_570nm_ was determined using a BioTek ELX808 plate reader.

### 2.8 Antigen processing and presentation

DCs were plated in R10 medium at 5 × 10^4^ cells per well of a 96 well, flat bottomed tissue culture plate with an equal number of the 1C5.1 T‐cell hybridoma, specific for the 46‐61 epitope of hen egg lysozyme (HEL) in the context of H‐2A^k^.^36^ Control wells contained DCs fixed for 5 minutes in 1% paraformaldehyde. Cultures were incubated for 18 hours with a serial 2‐fold dilution of whole HEL from a top concentration of 500 μg/mL. Concentrations of IL‐2 in culture supernatants were determined by sandwich ELISA, according to the manufacturer's instructions (eBioscience Mouse IL‐2 [Cat# 88‐7024‐88] ELISA Ready‐SET‐Go! kit).

### 2.9 Mixed leukocyte reaction (MLR)

Allogeneic T cells were enriched from dissociated C57BL/6 mouse spleens by passage over nylon wool. Purified T cells were labeled with CFSE using CellTrace CFSE Cell Proliferation Kit (Invitrogen, Cat# C34554) according to manufacturer's instructions and plated at 2 × 10^5^ cells per well of a 96 well round bottomed tissue culture plate. DCs were harvested and incubated in 10 μg/mL of MMC for 30 minutes, washed and titrated into cultures in triplicate from a top density of DCs:T cells of 1:4. Cocultures were incubated for 4 days before being blocked and counter‐stained with anti‐CD4‐APC mAb as described above. CD4^+^ T cell proliferation was analyzed using a FACSCalibur cytometer.

### 2.10 T cell activation in vivo

Female ipDC differentiated from iPSC_MEF_ (iPSC_MEF_‐DC) were harvested and pulsed with 100 nM Dby_479‐493_ peptide in PBS for 30 minutes. Male ipDC differentiated from iPSC_DC_ (iPSC_DC_‐DC) and peptide‐pulsed female iPSC_MEF_‐DC were resuspended in PBS and 4 × 10^6^ cells injected into the tail veins of female A1.RAG1^−/−^ mice in a 200 μL volume. PBS alone was used as a control. Spleens were harvested 20 hours later and dissociated into a single cell suspension. Erythrocytes were lysed and the splenocytes blocked and stained with anti‐CD4‐APC (BD Pharmingen, Cat# 553051) and anti‐CD69‐FITC (BD Pharmingen, Cat# 553236) mAbs as described above.

### 2.11 Statistical analysis

All presented data are representative of multiple independent experiments. Data are reported as mean ± SD. Results were analyzed using GraphPad Prism (Version 7.0). Where appropriate, results between groups were compared using a two‐tailed unpaired *t* test. Differences between groups were considered statistically significant at *P* < .05. Asterisks indicate the level of statistical significance (*P < .05; **P < .001).

## 3 RESULTS

### 3.1 Conventional ipDCs display a fetal phenotype

**Figure 1:**
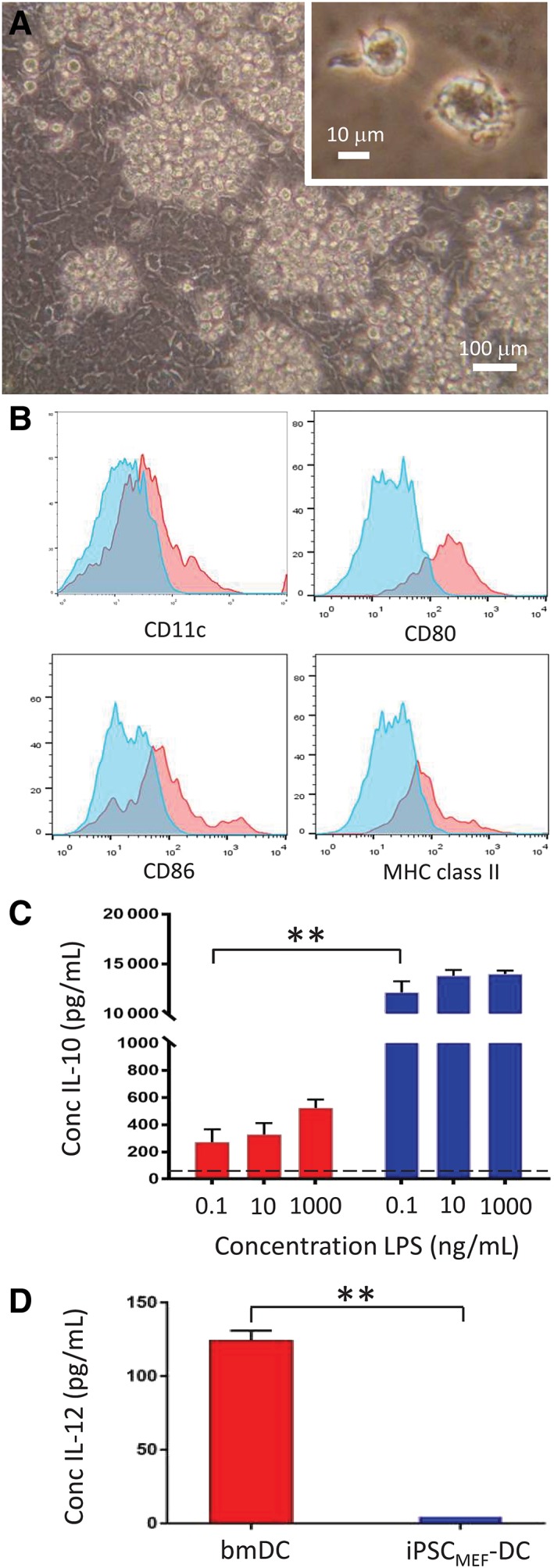
iPSC_MEF_‐dendritic cells (DCs) display a primitive, fetal phenotype. A, Phase‐contrast photomicrograph of iPSC_MEF_‐DCs developing in clusters around the perimeter of embryoid bodies (EBs). Inset: High magnification (×40) of individual iPSC‐derived dendritic cells (ipDCs). B, Flow cytometric analysis of iPSC_MEF_‐DCs, showing expression of CD11c, CD80, CD86, and MHC class II (red histograms). Blue histograms represent background staining with isotype‐matched control monoclonal antibodies (mAbs). C, IL‐10 secretion by bone marrow‐derived DCs (bmDCs) and iPSC_MEF_‐DCs in response to challenge with lipopolysaccharide (LPS). D, Secretion of IL‐12 by bmDCs and iPSC_MEF_‐DCs in response to challenge with 1 μg/mL LPS. C, D, Red bars denote bmDCs, blue bars represent iPSC_MEF_‐DCs. All data are representative of multiple experiments and are reported as mean values with error bars representing SD. **P < .001

To confirm previous observations of the fetal phenotype expressed by DCs differentiated from conventional iPSCs, MEFs were transduced with SeV vectors encoding Oct4, Sox2, Klf4, and cMyc and multiple monoclonal iPSC lines were established. The representative cell line, iPSC_MEF_SV_2_, displayed characteristic cell surface (SSEA‐1) and intracellular (Oct4, Nanog) markers of pluripotency (Supporting Information Figure S1A) and gave rise to EBs which, when engrafted under the kidney capsule of syngeneic recipients, formed teratomas containing tissues derived from each of the embryonic germ layers, confirming their pluripotency (Supporting Information Figure S1B). Culture of EBs in vitro with rmIL‐3 and GM‐CSF, as described previously,^35^ resulted in the emergence of DCs over a 14 day period with typical veiled morphology (Figure 1A). Phenotypic analysis of iPSC_MEF_‐DC demonstrated low levels of expression of CD11c, CD80, and CD86 co‐stimulatory molecules, and comparable low expression of MHC class II (Figure 1B), as previously observed.^37^ In response to LPS stimulation, iPSC_MEF_‐DCs secreted the anti‐inflammatory cytokine IL‐10, at levels approximately 27‐fold greater at the highest dose (1 μg/mL) compared to control bmDCs cultured in parallel (Figure 1C). In marked contrast, iPSC_MEF_‐DCs failed to secrete significant levels of IL‐12, unlike their bone marrow‐derived counterparts (Figure 1D), findings which are highly reminiscent of human DCs of fetal or neonatal origin.^13^, ^14^

### 3.2 CD11c‐purified source DCs display classical morphology, phenotype, and function

**Figure 2:**
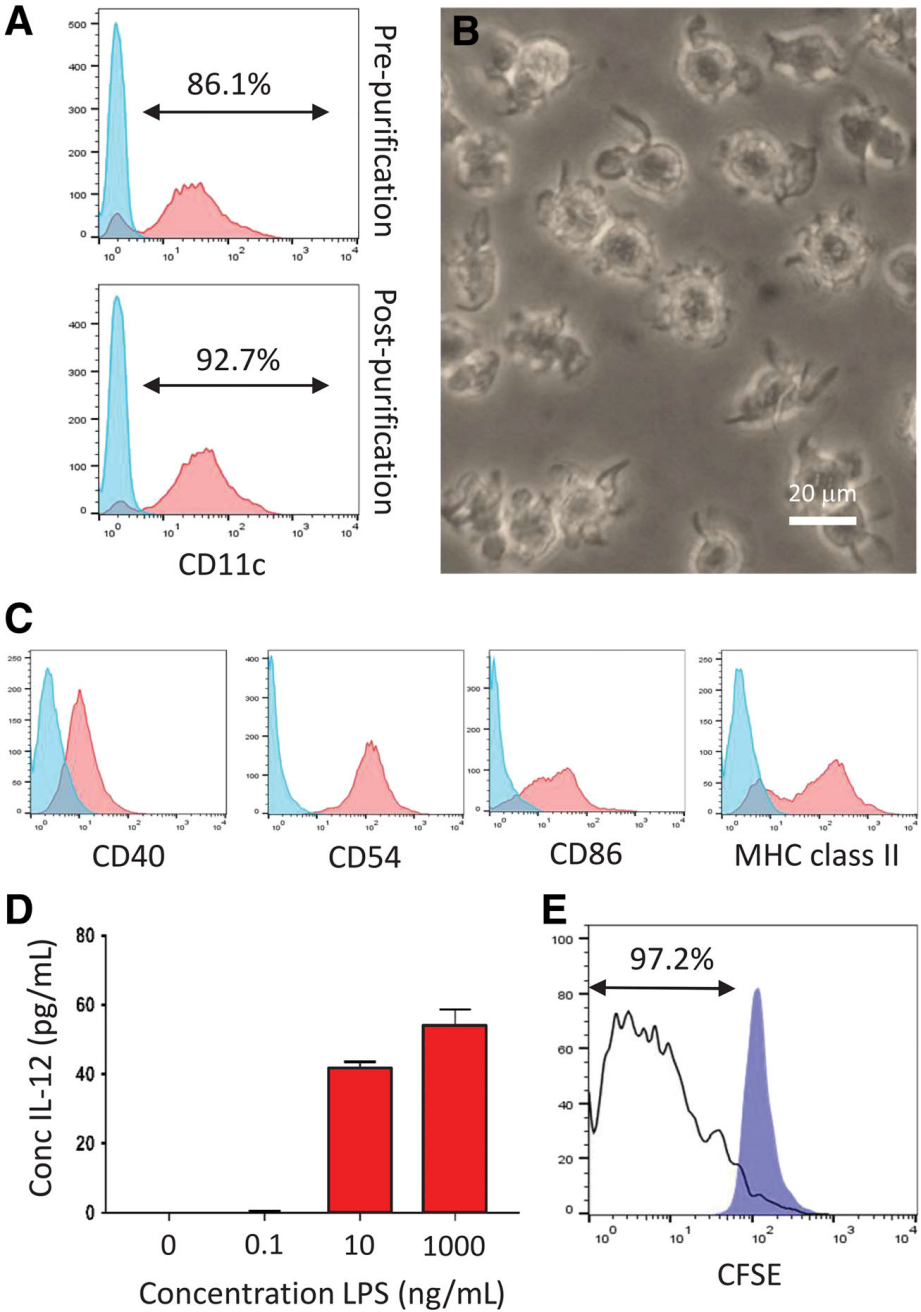
Characterization of source bone marrow‐derived dendritic cells (bmDCs) prior to reprogramming. A, CD11c expression of cells (red histograms) harvested at day 7 from cultures of bone marrow prior to and following the purification of DCs using CD11c‐labeled magnetic beads. Blue histograms represent background staining with an isotype control mAb. B, Phase‐contrast photomicrograph of bmDCs following purification showing typical veiled and dendritic morphology. C, Flow cytometric analysis of CD11c‐purified bmDCs showing expression of CD40, CD54, CD86, and MHC class II (red histograms). Blue histograms represent background staining with appropriate isotype controlled mAbs. D, IL‐12 secretion by CD11c‐purified bmDCs in response to increasing concentrations of lipopolysaccharide (LPS). All data reported as mean values with error bars representing SD of replicates. E, Immunostimulatory capacity of source bmDCs as a function of the proliferation of allogeneic T cells assessed by progressive dilution of CFSE. The unfilled histogram represents cocultures of DCs and allogeneic T cells whereas the blue histogram denotes unstimulated control T cells. All data are representative of multiple independent experiments

In order to investigate the feasibility of reprogramming primary DCs to pluripotency, an abundant source was cultured from bone marrow progenitors (see Materials and Methods section) and characterized prior to SeV transduction. CD11c^+^ cells were enriched to greater than 92% purity by passage over magnetic beads (Figure 2A), subsequent repeat experiments achieving a purity greater than 95%. CD11c^+^ cells displayed veiled morphology (Figure 2B) and expression of CD40, CD54, CD86, and MHC class II (Figure 2C) whereas exposure to LPS resulted in secretion of IL‐12 in a dose‐dependent manner (Figure 2D). Consistent with their phenotype, source bmDCs potently stimulated the proliferation in vitro of allogeneic, CFSE‐labeled T cells, 97.2% of cells having passed through one or more cell divisions during a 4‐day culture period (Figure 2E). Prior to reprogramming, source cells therefore displayed the desired phenotype and function of highly immunogenic DCs.

### 3.3 Fully differentiated DCs can be reprogrammed to pluripotency

**Figure 3:**
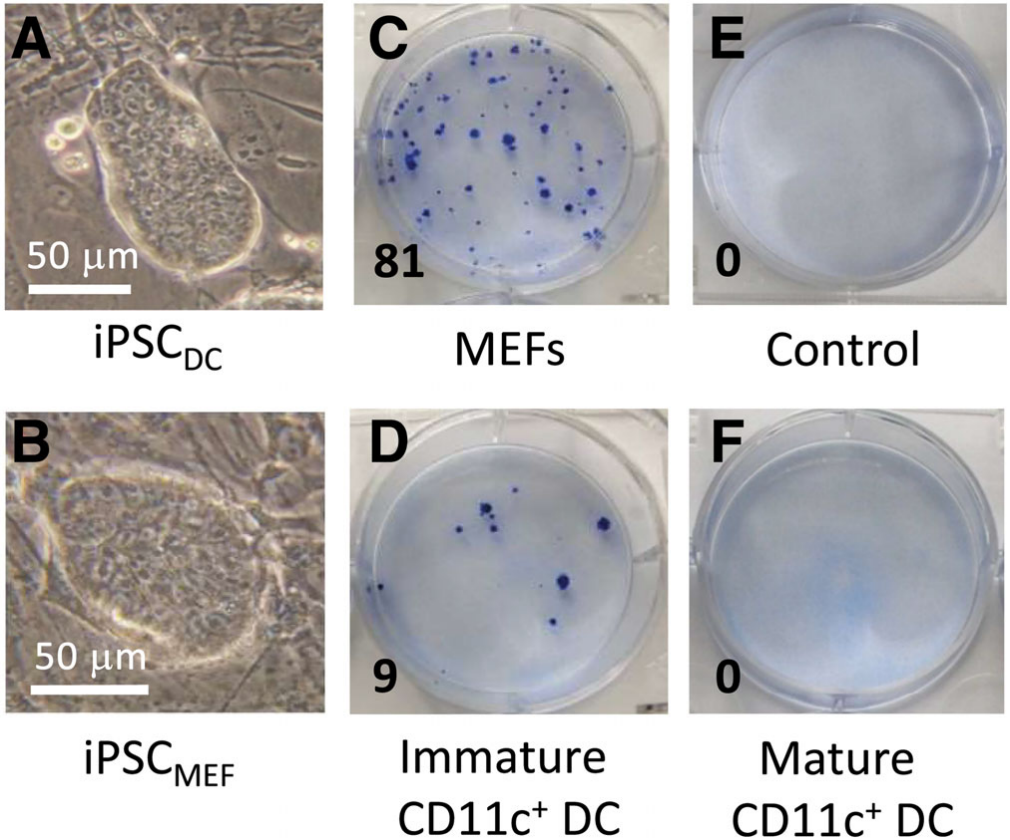
Reprogramming of bone marrow‐derived dendritic cells (bmDCs) to pluripotency. A, Phase‐contrast photomicrograph of a typical colony of induced pluripotent stem cells (iPSCs) reprogrammed from bmDCs (iPSC_DC_) at passage 7, cultured on mouse embryonic fibroblast (MEF) feeder cells. B, Phase‐contrast photomicrograph of a typical colony of iPSCs reprogrammed from MEFs (iPSC_MEF_), cultured on feeder cells, likewise at passage 7. C‐F, Methylene blue staining of six well plates revealing the abundance of iPSC colonies from a representative experiment in which equivalent numbers of MEFs (C) and bmDCs (D and F) were reprogrammed in parallel. E, Control well of MEFs without the addition of reprogramming vectors. F, Well containing equivalent numbers of bmDCs induced to mature in response to lipopolysaccharide (LPS) prior to reprogramming. Numbers in the bottom left hand corner of each image denote the number of iPSC colonies observed

Purified bmDCs were reprogrammed to pluripotency with the use of CytoTune 2.0 (SeV), forming iPSC‐like colonies as early as day 8 following transduction (Figure 3A). The greatest reprogramming efficiency was achieved using high levels of Klf4 (a ratio of KOS:cMyc:Klf4 of 5:5:5). Control MEFs, reprogrammed in parallel, likewise formed classical, domed iPSC colonies when plated onto monolayers of feeder cells in 6 well plates (Figure 3B). To quantify reprogramming efficiency, wells were stained with methylene blue to facilitate the enumeration of colonies. Reprogramming efficiency of control MEFs, a cell type particularly amenable to reprogramming, ranged between 0.18% and 1.68% (Figure 3C and Table 1). In contrast, the efficiency of DC reprogramming was significantly lower, ranging between 0.03% and 0.11% of cells successfully forming iPSCs (Figure 3D and Table 1). No colonies were observed in wells cultured in the absence of SeV (Figure 3E). Likewise, CD11c^+^ bmDCs induced to mature by prior exposure to LPS, failed to yield any iPSC colonies (Figure 3F), suggesting that the tractability of DCs for reprogramming is highly dependent on their maturation status.

**Table 1:** Comparison of reprogramming efficiency of mouse embryonic fibroblasts (MEFs) and bone marrow‐derived dendritic cells (bmDCs)

	Experiment 1		Experiment 2		Experiment 3	
	MEFs	bmDCs	MEFs	bmDCs	MEFs	bmDCs
Mean # cells plated per well	5.0 × 10^3^	2.5 × 10^4^	4.4 × 10^4^	8.8 × 10^3^	4.0 × 10^5^	3.3 × 10^4^
Mean # colonies per well	84.0	7.2	77.0	9.4	326.0	26.0
Reprogramming efficiency (%)	1.68	0.03	0.18	0.11	0.82	0.08

**Figure 4:**
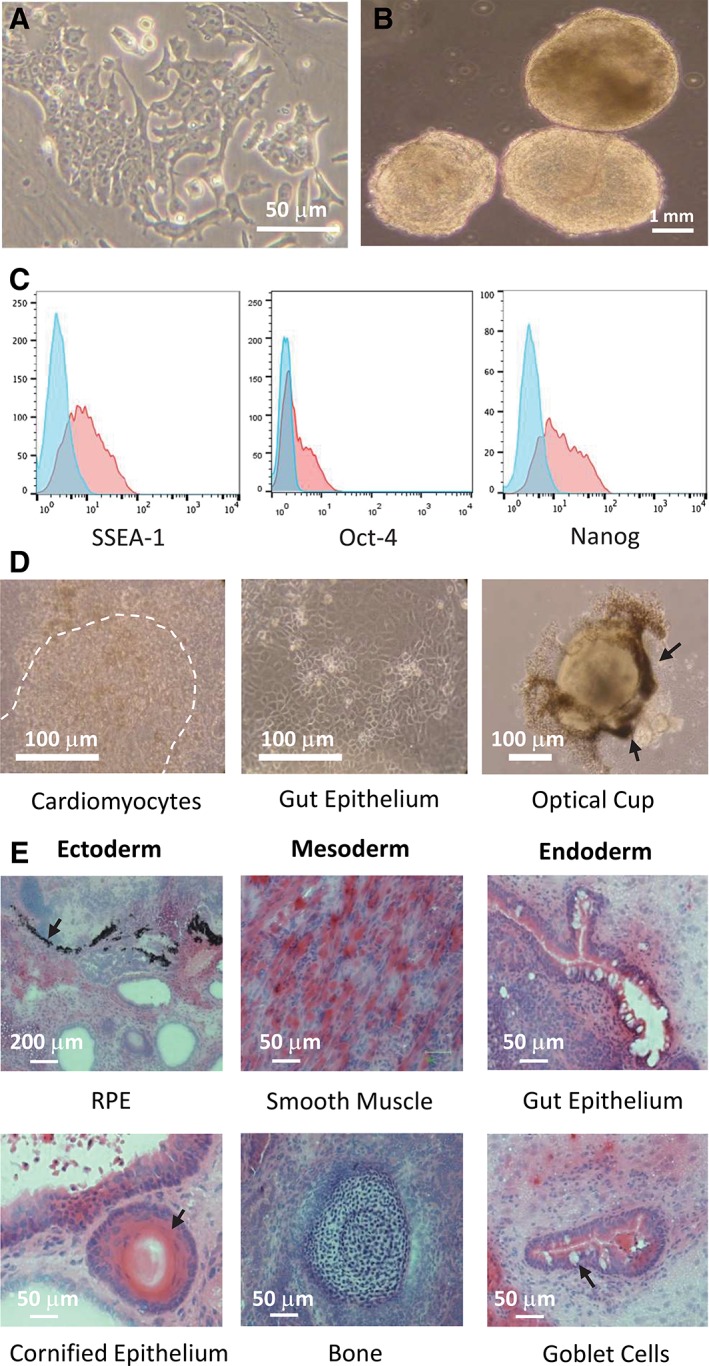
Characterization of iPSC_DC_ demonstrating that full pluripotency can be achieved from source bone marrow‐derived dendritic cells (bmDCs). A, Phase‐contrast photomicrograph of a typical iPSC_DC_ colony cultured on gelatin‐coated flasks demonstrating characteristic nuclear morphology and the prominent nucleoli, typical of pluripotent cells. B, Photomicrograph of EBs at day 10 of culture, derived from iPSC_DC_ at passage 6. Approximately 34% of EBs were found to contract spontaneously in suspension culture (see Supplementary Online Video S1). C, Flow cytometric analysis of iPSC_DC_ demonstrating expression of the pluripotency markers SSEA‐1, Oct4, and Nanog (red histograms). Blue histograms represent isotype‐matched control mAbs. D, Phase‐contrast photomicrographs of tissues differentiated in vitro from iPSC_DC_, showing tissues derived from each of the three embryonic germ layers: mesoderm (cardiomyocytes: the broken line denotes the region of spontaneously contracting tissue), endoderm (gut epithelium), and ectoderm (optic cup identified by the preponderance of retinal pigmented epithelial cells, arrows). E, Hematoxylin and eosin (H&E) stained histological sections of teratomas formed following the engraftment of EBs derived from iPSC_DC_ under the kidney capsule of syngeneic mice. Photomicrographs show examples of tissues representing the three embryonic germ layers: Ectoderm (retinal pigment epithelium, cornified epithelium [arrows]), mesoderm (smooth muscle, bone), and endoderm (gut epithelium, goblet cells [arrow])

Although reprogramming efficiency of bmDC was comparatively low, numerous colonies were harvested and monoclonal iPSC lines established. The representative iPSC_DC_ cell line (iPSC_DC_SV_C_) formed typical colonies on gelatin (Figure 4A) and readily formed EBs in suspension culture (Figure 4B) at all passages examined (passages 5‐20). Interestingly, EBs derived from early passages of iPSC_DC_ contained a significant proportion that contracted rhythmically in suspension (mean 34.0% ± 6.55%) suggesting a preponderance of cardiomyocytes due to an inherent preference for differentiation along the mesodermal lineage (Supplemental Online Video S1). In contrast, no rhythmic contractions of EBs derived from passage‐matched iPSC_MEF_ were ever observed. Karyotypic analysis of iPSC_DC_ at passage 7 demonstrated the median number of chromosomes to be 40XY, confirming the line to be male and lacking gross karyotypic abnormalities: although at this passage, qPCR revealed the persistence of SeV reprogramming vectors and transgenes, these were found to be completely eradicated by passage 11 (data not shown). Flow cytometric analysis confirmed expression of markers of pluripotency including SSEA‐1, Oct‐4, and Nanog (Figure 4C). To determine the differentiation capacity of iPSC_DC_, a more stringent measure of pluripotency, day 14 EBs were plated onto tissue culture plastic and permitted to differentiate in an undirected manner. The resulting cultures included derivatives of mesoderm (cardiomyocytes) and endoderm (gut epithelium), as well as fully formed optic cups as evidence of ectodermal differentiation, readily identified by the abundance of retinal pigmented epithelial cells (Figure 4D, arrows). Finally, day 14 iPSC_DC_‐derived EBs engrafted under the kidney capsule of syngeneic mice formed teratomas in vivo likewise containing tissues derived from each of the three germ layers upon histological analysis (Figure 4E), demonstrating unequivocally that bmDCs are amenable to reprogramming with SeV, forming fully pluripotent iPSC lines.

### 3.4 iPSC_DC_‐DCs retain the immunogenic phenotype of source bmDCs

**Figure 5:**
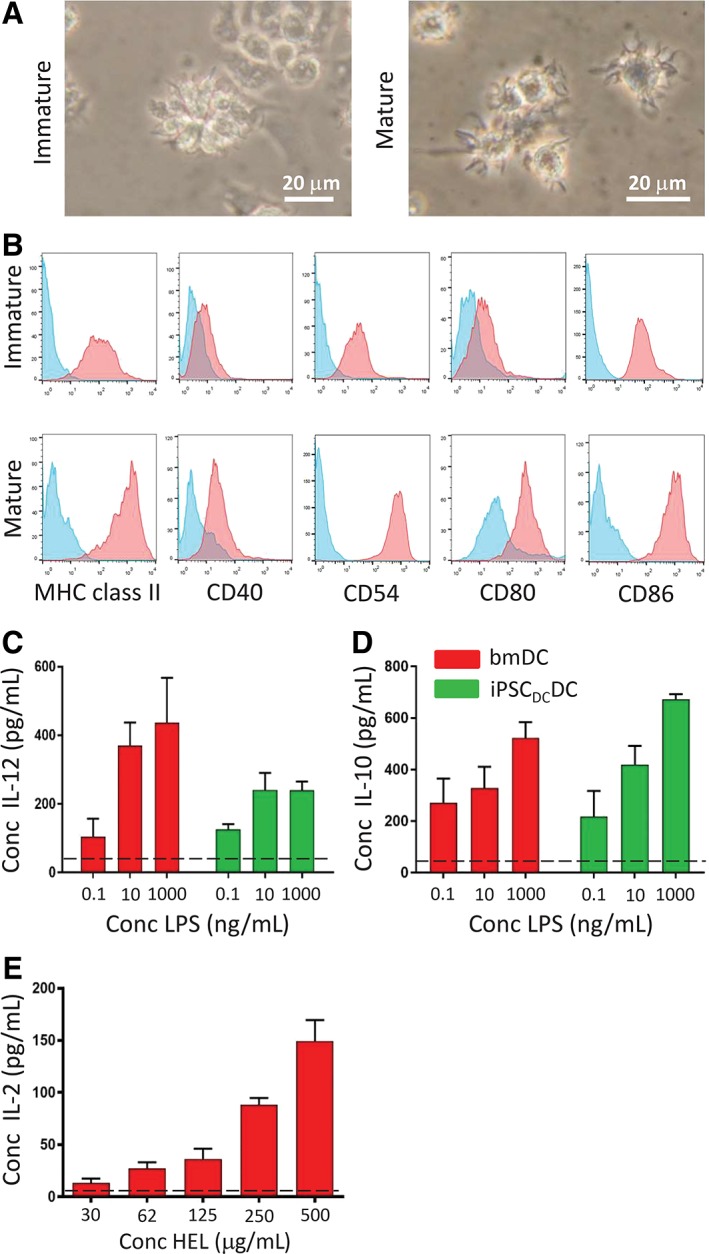
Characterization of iPSC‐derived dendritic cells (ipDCs) differentiated from iPSC_DC_. A, Phase contrast photomicrographs of ipDCs differentiated from iPSC_DC_ prior to maturation (left hand image) and following maturation in response to lipopolysaccharide (LPS) (right hand image). B, Flow cytometric analysis of early passage iPSC_DC_‐DC, demonstrating a baseline expression of MHC class II, CD40, CD54, CD80, and CD86 (red histograms), and their upregulation following coculture with 1 μg/mL LPS. Blue histograms represent background staining with appropriate isotype‐matched control mAbs. C, Comparison of IL‐12 secretion by bone marrow‐derived dendritic cells (bmDCs) and iPSC_DC_‐DCs at passage 5 in response to challenge with LPS. Background levels of IL‐12 in the absence of LPS are shown as a broken line. D, Comparison of IL‐10 secretion by bmDCs and iPSC_DC_‐DCs passage 5 in response to LPS. The dashed line denotes the background levels of IL‐10 detected in the absence of LPS. E, Processing and presentation of HEL by iPSC_DC_‐DCs measured as a function of IL‐2 secretion by the 1C5.1 T‐cell hybridoma, specific for HEL_46‐61_/H‐2A^k^. All data are representative of multiple experiments and represent mean values with error bars denoting SD

EBs derived from iPSC_DC_ were plated onto tissue culture plastic in medium supplemented with rmIL‐3 and GM‐CSF to initiate DC differentiation. Abundant clusters of ipDCs appeared predominately around the perimeter of EBs from day 4 of culture onwards (Figure 5A) and were harvested for characterization around day 16. Unlike their iPSC_MEF_‐derived counterparts, early passage iPSC_DC_‐DCs expressed a profile highly reminiscent of the immunogenic phenotype of the source bmDCs, including expression of CD40, CD54, CD80, and CD86 as well as significant MHC class II (Figure 5B). Co‐stimulatory molecules and MHC class II were consistently upregulated upon exposure to 1 μg/mL LPS to induce maturation (Figure 5B), which also gave rise to cells with highly dendritic morphology (Figure 5A). Importantly, levels of IL‐12 secretion in response to LPS were not significantly different from those obtained from bmDCs, even at the highest concentration of LPS used (*P* = .06) (Figure 5C), which contrasted strongly with the secretion profile of conventional ipDCs differentiated from iPSC_MEF_ (Figure 1D). Levels of IL‐10 were likewise largely indistinguishable from those of bmDCs cultured in parallel (Figure 5D).

In order to determine the capacity of iPSC_DC_‐DCs to process and present exogenous protein antigen in the context of MHC class II, purified cells were pulsed with varying concentrations of HEL and cocultured with the 1C5.1 T‐cell hybridoma, specific for the 46‐61 epitope presented in the context of H‐2A^k^.^36^ T cell activation, measured as a function of IL‐2 secretion, was observed in a dose dependent manner (Figure 5E) and was inhibited by prior fixation of the iPSC_DC_‐DCs to prevent antigen uptake (broken line). These findings therefore suggest that early passage iPSC_DC_‐DCs exhibit the phenotypic and functional integrity of the source bmDCs from which the parent iPSC line was originally derived.

### 3.5 iPSC_DC_‐DCs display a similar differentiation efficiency to iPSC_MEF_‐DCs but circumvent the fetal phenotype

**Figure 6:**
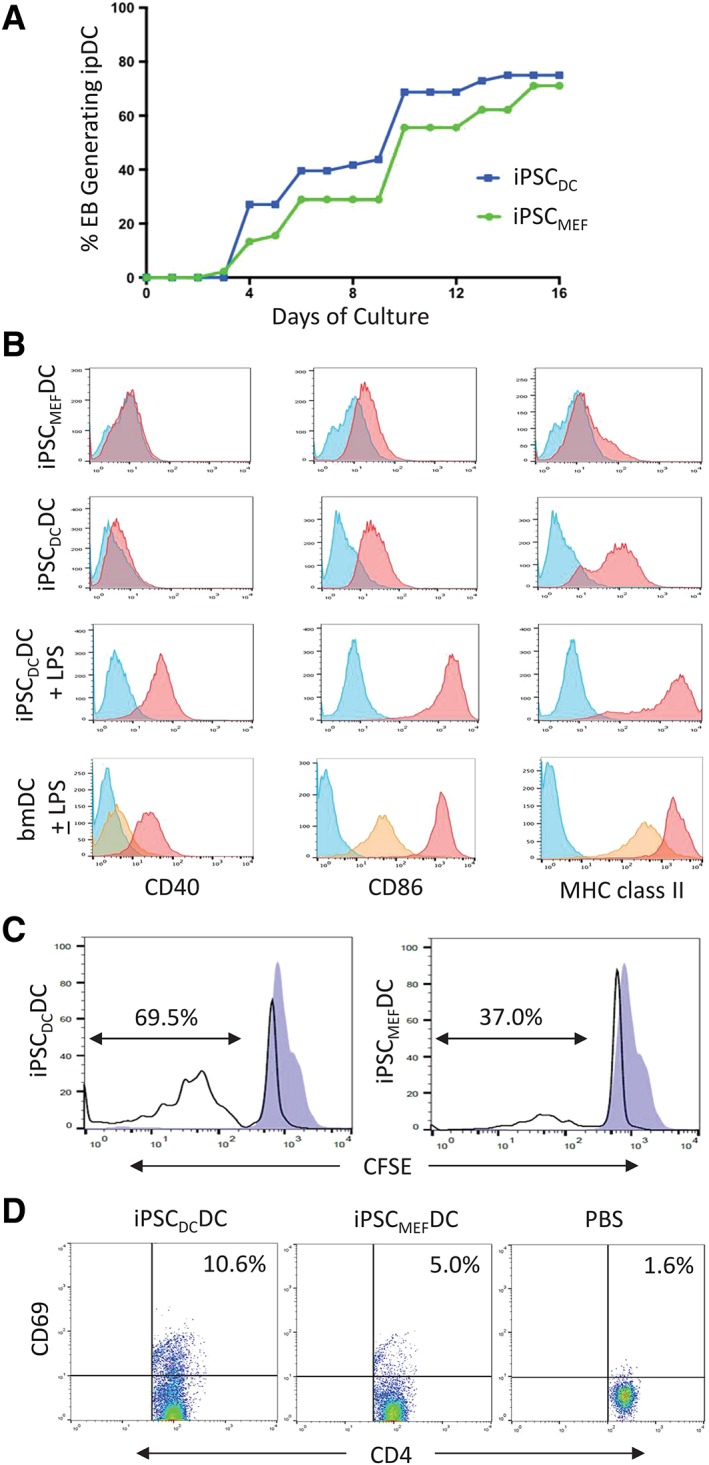
iPSC‐derived dendritic cells (ipDCs) differentiated from iPSC_DC_ display an adult phenotype compared to their iPSC_MEF_‐derived counterparts. A, Proportion of EBs derived from iPSC_DC_ and iPSC_MEF_ (both at passage 7) capable of generating ipDCs over a 16 day culture period. B, Flow cytometric analysis of CD40, CD86, and MHC class II expression (red histograms) by iPSC_MEF_‐DCs, iPSC_DC_‐DCs, and control bone marrow‐derived dendritic cells (bmDCs either immature or following maturation in response to lipopolysaccharide (LPS). Yellow histograms represent staining of immature bmDCs; blue histograms denote background staining with isotype‐matched control mAbs. C, Comparison of the immunostimulatory capacity of ipDCs differentiated from iPSC_DC_ and iPSC_MEF_ measured as a function of the proliferation of CFSE‐labeled allogeneic T cells (unfilled histograms). Blue histograms represent control T cells cultured in the absence of DCs. D, in vivo stimulation of CD4^+^ T cells following the i.v. administration of iPSC_DC_‐DCs, iPSC_MEF_‐DCs, or phosphate‐buffered saline (PBS) alone to female A1.RAG1^−/−^ mice. T cell activation is shown as a function of the upregulation of CD69, an early activation marker 20 hours after administration. All data are representative of multiple independent experiments

Phenotypic and functional properties of iPSC_DC_‐DCs and iPSC_MEF_‐DCs were directly compared following their differentiation from developmentally matched iPSC lines at passage 7 of culture. Both iPSC_DC_ and iPSC_MEF_ produced abundant ipDCs over a similar time‐course, although iPSC_DC_‐DCs were observed 1 day earlier than iPSC_MEF_‐DCs (Figure 6A), a finding that was consistent for all passages examined. The difference in proportion of EBs from iPSC_DC_ and iPSC_MEF_ successfully producing ipDCs was not statistically significant for any passage (*P* = .33). Although flow cytometric analysis demonstrated a similar level of CD40 expression in immature bmDCs, iPSC_DC_‐DCs, and iPSC_MEF_‐DCs, immature bmDCs and iPSC_DC_‐DCs displayed higher baseline levels of CD86 and MHC class II expression than their iPSC_MEF_‐derived counterparts, both of which were dramatically upregulated following exposure to LPS (Figure 6B): the phenotype of iPSC_DC_‐DCs was, therefore, largely indistinguishable from source bmDCs. To investigate whether differences in surface phenotype of iPSC_DC_‐DCs and iPSC_MEF_‐DCs had functional consequences, either population was cocultured with purified CFSE‐labeled allogeneic T cells. After 4 days of culture, 67.0% of T cells incubated with iPSC_DC_‐DCs had passed through one or more cell divisions compared to 36.7% of cells cocultured with iPSC_MEF_‐DCs (Figure 6C). In order to examine their immunogenicity in vivo, either population of DCs was administered i.v. to female A1.RAG1^−/−^ mice expressing a transgenic TCR specific for Dby_479‐493_/H‐2E^k^, endogenously presented by ipDCs. After 20 hours, splenocytes were harvested and analyzed for upregulation of the early activation marker CD69. In comparison to PBS controls, both populations stimulated activation of CD4^+^ T cells, although a greater proportion were stimulated by iPSC_DC_‐DCs (10.6%) compared to iPSC_MEF_‐DCs (5.0%) (Figure 6D). Experiments comparing ipDCs from passage‐matched iPSC_DC_ and iPSC_MEF_ revealed the immunogenic phenotype of iPSC_DC_‐DCs to be retained for 15 passages of the parent cell line after which expression of MHC class II and co‐stimulatory molecules became more variable, ultimately reverting to a fetal phenotype, indistinguishable from ipDCs differentiated from iPSC_MEF_ (Supporting Information Table S1). Together, these data support the notion that early passage iPSCs reprogrammed from primary DCs readily redifferentiate into DCs that are not subject to the fetal phenotype that typifies those differentiated from conventional iPSC_MEF_ and therefore display an immunostimulatory phenotype conducive to their use in immunotherapy.

## 4 DISCUSSION

The importance of DCs in the initiation of immune responses among antigen‐naïve T cells has fueled interest in iPSCs as a renewable and scalable source of rare DC subsets with desirable properties not normally vested in moDCs,^3^, ^4^ the population most frequently used in clinical trials.^2^ Nevertheless, in common with other cell types differentiated from iPSCs, ipDCs are arrested at an early stage of development more closely resembling fetal or neonatal DCs, as evidenced by their low expression of MHC class II and co‐stimulatory molecules and failure to secrete IL‐12 in response to PRR agonists (Figure 1B,D), a phenotype equally evident among ipDCs of mouse^37^ and human origin.^5^, ^12^ Given the adverse impact such a phenotype may have on their downstream use in immunotherapy, we have sought to address these limitations by exploiting the epigenetic memory of iPSCs for the cell type from which they were originally reprogrammed, hypothesizing that the epigenetic signature of iPSCs reprogrammed from existing DCs might support the subsequent differentiation of highly immunogenic ipDCs, suitable for vaccination purposes.

Using CD11c‐purified mouse bmDCs as proof of principle (Figure 2), we have demonstrated that immature terminally differentiated DCs may be reprogrammed to pluripotency with the use of SeV vectors for the delivery of Oct4, Sox2, Klf4, and c‐Myc transcription factors. Successful reprogramming was demonstrated by cell surface expression of SSEA‐1, combined with intracellular expression of Nanog and Oct4 (Figure 4C)^38^, ^39^, ^40^, ^41^, ^42^ and full pluripotency formally verified in teratoma assays in which iPSC_DC_ were shown to differentiate in vivo into multiple tissues derived from each of the three embryonic germ layers (Figure 4E). These findings were unexpected given the sentinel function performed by DCs that is responsible for their sensitivity to viral pathogens and pathogen‐associated molecular patterns (PAMPs), such as LPS. Indeed, exposure to LPS is known to initiate a program of maturation followed ultimately by cell death,^43^, ^44^ providing a likely explanation for our inability to achieve the reprogramming of mature bmDCs to pluripotency (Figure 3F). Although others have demonstrated that DCs have increased susceptibility to apoptosis following viral infection,^45^, ^46^ we have demonstrated that terminally differentiated bmDCs can survive the process of viral transduction, and are tractable candidates for reprogramming to pluripotency, having generated numerous iPSC colonies in each of 8 consecutive independent experiments, yielding an average reprogramming efficiency of 0.07%. Given the nonintegrating nature of SeV vectors, together with the transient expression of the reprogramming factors, SeV transduction may be an appropriate approach to the generation of therapeutic grade iPSCs from DCs,^47^ although other approaches, such as the use of mRNA that leaves no genomic footprint and persists only transiently, may ultimately prove more compatible with downstream clinical use.

Although various cell types reprogrammed to pluripotency have shown a greater propensity to redifferentiate into the cell type of origin than distantly related cell types,^31^, ^32^ not all such attempts have proven successful. For example, Wada and colleagues generated iPSCs from murine B cells but were subsequently unable to direct their differentiation along the B cell lineage pathway in vitro.^48^ In contrast, iPSC_DC_ showed enhanced potential for redifferentiation toward a mesodermal fate, as evidenced by a significant proportion of EBs (34%) contracting rhythmically in suspension (Figure 4B, Supplemental Online Video S1), consistent with abundant cardiomyocyte commitment: whereas areas of beating cardiomyocytes were routinely noted among EBs from iPSC_MEF_ once plated onto tissue culture plastic, the spontaneous contraction of whole EBs in suspension culture had not previously been observed. Furthermore, upon addition of IL‐3 and GM‐CSF, iPSC_DC_ readily generated abundant ipDCs displaying a phenotype, function and responsiveness to maturation stimuli typical of adult rather than fetal DCs (Figure 5), indeed this source was largely indistinguishable from the bmDCs from which the iPSCs were originally derived. Although this phenotype conferred greater immunogenicity on ipDCs than their counterparts differentiated from iPSC_MEF_ (Figure 6C,D), their expression of MHC class II and co‐stimulatory molecules became erratic from passage 15 onward, the proportion of ipDCs expressing MHC class II decreasing from 74.8% at passage 13% to 0.17% at passage 15 (Supporting Information Table S1). Furthermore, the decrease in proportion of cells expressing co‐stimulatory molecules such as CD86 from 79.6% to 11.9% is consistent with reversion to a more fetal phenotype, presumably as the epigenetic memory of the source DCs is progressively erased. Nevertheless, if translatable to cells of human origin, retention of the epigenetic memory of the source DCs for multiple passages after the full clearance of the viral vectors, provides a window of opportunity for the generation of highly immunogenic DCs suitable for immunotherapeutic purposes. Importantly, this therapeutic window may be significantly extended through the use of recently described SeV vectors targeted by microRNA‐302, known to be rapidly upregulated following the reprogramming of cells to pluripotency. This elegant system results in the auto‐erasure of vectors and transgenes shortly after the establishment of iPSCs of either mouse or human origin.^49^ In this context, we and others^50^ have recently demonstrated the feasibility of reprogramming human moDCs to pluripotency suggesting the potential for the future clinical translation of our findings.

In terms of cancer immunotherapy, our approach may permit the derivation of iPSC lines from small numbers of moDCs differentiated in vitro from a patient's peripheral blood monocytes. This approach carries many advantages over the conventional use of dermal fibroblasts. First, the isolation of monocytes and their subsequent differentiation may be achieved over a 2 day period,^51^ comparing favorably with the duration of time required to expand dermal fibroblasts from a skin biopsy to sufficient numbers required for reprogramming.^52^ In addition, simple venipuncture required for monocyte isolation is significantly less invasive, thereby carrying a lower risk of infection compared to skin punch biopsies. Furthermore, due to their superficial location, dermal fibroblasts are more susceptible to the accumulation of mutations from environmental insults such as UV radiation which become enshrined in the resulting iPSCs, having potential consequences for downstream therapeutic applications. Conversely, monocytes are continuously regenerated from a small pool of genetically stable hematopoietic stem cells, which are largely protected from environmental mutagens by virtue of their quiescence. iPSCs generated from moDCs are likely, therefore, to inherit a genome largely untainted by mutation. Finally, as demonstrated here, the use of moDCs as a starting population is likely to capture the epigenetic signature of DCs within early passage iPSCs, permitting the generation of mature immunostimulatory ipDCs for vaccination purposes.

## 5 CONCLUSION

Exploiting iPSCs as a novel source of DC subsets for use in immunotherapy is predicated on circumventing the limitations of the fetal phenotype they display which may prove an impediment to their downstream clinical application. Through the simple expedient of reprogramming existing DCs to pluripotency, our findings suggest that the epigenetic profile of the resulting iPSC lines supports the differentiation of abundant immunostimulatory DCs providing a scalable source of otherwise inaccessible subsets for vaccination purposes.

## CONFLICT OF INTEREST

P.J.F., T.D., and C.H. jointly own intellectual property relevant to the research reported here. The other authors indicated no financial relationships.

## AUTHOR CONTRIBUTIONS

C.H.: conception and design, collection of data, data analysis and interpretation, manuscript writing; T.J.D., P.L.: Collection of data, data analysis and interpretation; P.S.: conception and design, data analysis and interpretation; P.J.F.: conception and design, financial support, manuscript writing, final approval of manuscript.

## Supporting information


**Supporting Information Table 1** Surface phenotype of DC differentiated from iPSC_DC_ showing dramatic reduction in expression of MHC class II and co‐stimulatory molecules at Passage 15 of the parent iPSC line, consistent with loss of the epigenetic memory of the DC of origin.

**Supporting Information Figure S1** Characterization of the control iPSC_MEF_ cell line, iPSC_MEF_SV2. **(A)**: Flow cytometric analysis, demonstrating expression of the pluripotency markers SSEA‐1, Oct4 and Nanog (red histograms). Blue histograms represent background staining with isotype‐matched control mAbs. **(B)**: Hematoxylin and eosin (H&E) stained sections of teratomas formed following the engraftment of EBs derived from iPSC_MEF_ under the kidney capsule of syngeneic mice. Photomicrographs show representative tissues from each the embryonic germ layers.

**Supplementary Video 1** Early passage iPSC_DC_ display tendency to differentiate into tissues of mesodermal origin. Video recording of a typical EB at day 10 of culture differentiated from iPSC_DC_ at passage 5. Rhythmical contractions of the EB suggest the presence of cardiomyocytes, consistent with the preferential redifferentiation of the cell line toward the mesodermal lineage.

## Data Availability

The data that support the findings of this study are available from the corresponding author upon reasonable request.
