# Supplementary material for: Induced pluripotent stem cells reprogrammed from primary dendritic cells provide an abundant source of immunostimulatory dendritic cells for use in immunotherapy
Source: Stem Cells. 2019 Oct 31;38(1):67–79. doi: 10.1002/stem.3095 (PMC7003857; doi:10.1002/stem.3095)
Supplement: Supplementary file 1 — Supporting Information Table 1 Surface phenotype of DC differentiated from iPSCDC showing dramatic reduction in expression of MHC class II and co‐stimulatory molecules at Passage 15 of the parent iPSC line, consistent with loss of the epigenetic memory of the DC of origin. [file STEM-38-67-s001.docx]

Horton *et al*. Supporting Information Table 1

|  | **% iPSC_DC_-DC Expressing Surface Markers** | | |
| --- | --- | --- | --- |
| **Passage of iPSC_DC_** | **CD80** | **CD86** | **MHC class II** |
| **Passage 7** | 35.5 | 40.4 | 41.7 |
| **Passage 9** | 42.4 | 83.6 | 84.4 |
| **Passage 13** | 62.6 | 79.6 | 74.8 |
| **Passage 15** | 11.9 | 11.9 | 0.17 |

**Supporting Information Table 1.** Surface phenotype of DC differentiated from iPSC_DC_ showing dramatic reduction in expression of MHC class II and co-stimulatory molecules at Passage 15 of the parent iPSC line, consistent with loss of the epigenetic memory of the DC of origin.
